# Long‐term cattle grazing shifts the ecological state of forest soils

**DOI:** 10.1002/ece3.8786

**Published:** 2022-03-31

**Authors:** Willem Proesmans, Christopher Andrews, Alan Gray, Rob Griffiths, Aidan Keith, Uffe N. Nielsen, David Spurgeon, Richard Pywell, Bridget Emmett, Adam J. Vanbergen

**Affiliations:** ^1^ Agroécologie, AgroSup Dijon INRAE Univ. Bourgogne Franche‐Comté Dijon France; ^2^ UK Centre for Ecology and Hydrology (UKCEH) Penicuik UK; ^3^ Environment Centre Wales UK Centre for Ecology and Hydrology (UKCEH) Bangor UK; ^4^ Lancaster Environment Centre UK Centre for Ecology and Hydrology (UKCEH) Bailrigg UK; ^5^ Hawkesbury Institute for the Environment Western Sydney University Penrith New South Wales Australia; ^6^ UK Centre for Ecology and Hydrology (UKCEH) Wallingford UK

**Keywords:** collembola, earthworms, forest grazing, oribatid and mesostigmatid mites, soil chemistry, soil microbes

## Abstract

Cattle grazing profoundly affects abiotic and biotic characteristics of ecosystems. While most research has been performed on grasslands, the effect of large managed ungulates on forest ecosystems has largely been neglected. Compared to a baseline seminatural state, we investigated how long‐term cattle grazing of birch forest patches affected the abiotic state and the ecological community (microbes and invertebrates) of the soil subsystem. Grazing strongly modified the soil abiotic environment by increasing phosphorus content, pH, and bulk density, while reducing the C:N ratio. The reduced C:N ratio was strongly associated with a lower microbial biomass, mainly caused by a reduction of fungal biomass. This was linked to a decrease in fungivorous nematode abundance and the nematode channel index, indicating a relative uplift in the importance of the bacterial energy‐channel in the nematode assemblages. Cattle grazing highly modified invertebrate community composition producing distinct assemblages from the seminatural situation. Richness and abundance of microarthropods was consistently reduced by grazing (excepting collembolan richness) and grazing‐associated changes in soil pH, Olsen P, and reduced soil pore volume (bulk density) limiting niche space and refuge from physical disturbance. Anecic earthworm species predominated in grazed patches, but were absent from ungrazed forest, and may benefit from manure inputs, while their deep vertical burrowing behavior protects them from physical disturbance. Perturbation of birch forest habitat by long‐term ungulate grazing profoundly modified soil biodiversity, either directly through increased physical disturbance and manure input or indirectly by modifying soil abiotic conditions. Comparative analyses revealed the ecosystem engineering potential of large ungulate grazers in forest systems through major shifts in the composition and structure of microbial and invertebrate assemblages, including the potential for reduced energy flow through the fungal decomposition pathway. The precise consequences for species trophic interactions and biodiversity–ecosystem function relationships remain to be established, however.

## Graphical abstract

Livestock grazing profoundly affects forest soil biotic and abiotic characteristics. We detected a grazer‐induced shift from the fungal to the bacterial energy channel, a strong increase in (anecic) earthworm biomass and a sharp decline in microarthropod abundance and species richness.

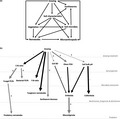

## 1 INTRODUCTION

Large ungulate herbivores can profoundly modify ecosystems and the structure of plant–animal assemblages (Stark et al., 2000; Wang et al., 2006; Wardle et al., 2001). Interest in cattle grazing of forest has increased recently due to benefits for nature conservation (Van Uytvanck et al., 2014) and trends toward agroforestry (Röhrig et al., 2020) and forest fire prevention (Ruiz‐Mirazo, 2011). Wood pasture and forest grazing by cattle are ancient forms of agriculture that shaped the European landscape from at least the Middle Ages up to the 19th century (Kirby et al., 1995), but which were almost completely abandoned in Northwest Europe due to changes in agricultural practices (Bomanowska & Kiedrzyński, 2011).

Biotic and abiotic responses to grazing are often idiosyncratic and highly context dependent, varying with climate (Semmartin et al., 2004), soil nutrient content (Bardgett & Wardle, 2003), ecosystem type (Andriuzzi & Wall, 2017), grazer identity and intensity (Andriuzzi & Wall, 2017; Eldridge et al., 2017). Selective grazing on vegetation may directly shape plant community structure (Hobbs et al., 1996; Rambo & Faeth, 1999) by suppressing competitive dominants and facilitating the succession of a distinctive plant community (Fowler, 2002; Pykälä, 2003; Rambo & Faeth, 1999) varying in net primary production (NPP) and nutrient concentrations with potential effects on soil‐dwelling decomposers (Wardle & Bardgett, 2004).

Herbivorous animals may shift soil nutrient dynamics from slow‐cycling, recalcitrant fungal decomposition pathways to faster nutrient turnover via excreta and bacterial decomposition, hence shortcutting the decomposition pathway (Bardgett & Wardle, 2003). These changes in nutrient cycling may have cascading effects on the soil food web (Wang et al., 2020), affecting microbes and soil fauna in complex, context‐dependent ways. Moreover, an increased level of disturbance (e.g., due to trampling), and resulting soil compaction, may reduce water holding capacity (Houlbrooke & Laurenson, 2013), affect root growth (Unger & Kaspar, 1994), alter microbial activity and biomass (Tan et al., 2005), and negatively affect soil mesofauna (Cole et al., 2008), which may affect nutrient cycling. While direct and indirect effects of ungulate grazing change diversity and abundance of most functional groups in the forest soil, it may also lead to a shift in community composition (Behan‐Pelletier, 1999). Increased nutrient cycling (Siepel, 1996) and physical disturbances (Cole et al., 2008) may cause shifts from long‐living K‐species to fast‐reproducing r‐species.

Compared to the large body of knowledge about the effects of cattle grazing on pasture soils (e.g., Clapperton et al., 2002; Wang et al., 2020; Yang et al., 2013), far less is known about the impact on forest ecosystems. Most studies focus on the effect on vegetation (Tasker & Bradstock, 2006; Van Uytvanck & Hoffmann, 2009) and forest regeneration (Fortuny et al., 2020) or assess the effects of wild browsers such as deer (Popma & Nadelhoffer, 2020). Moreover, while there is research from boreal (e.g., reindeer; Santalahti et al., 2018; Stark et al., 2000) and tropical forests (e.g., cattle; Stern et al., 2002), the effects of managed ungulates on forest soil diversity and functioning in temperate regions remain largely unexplored.

**FIGURE 1:**
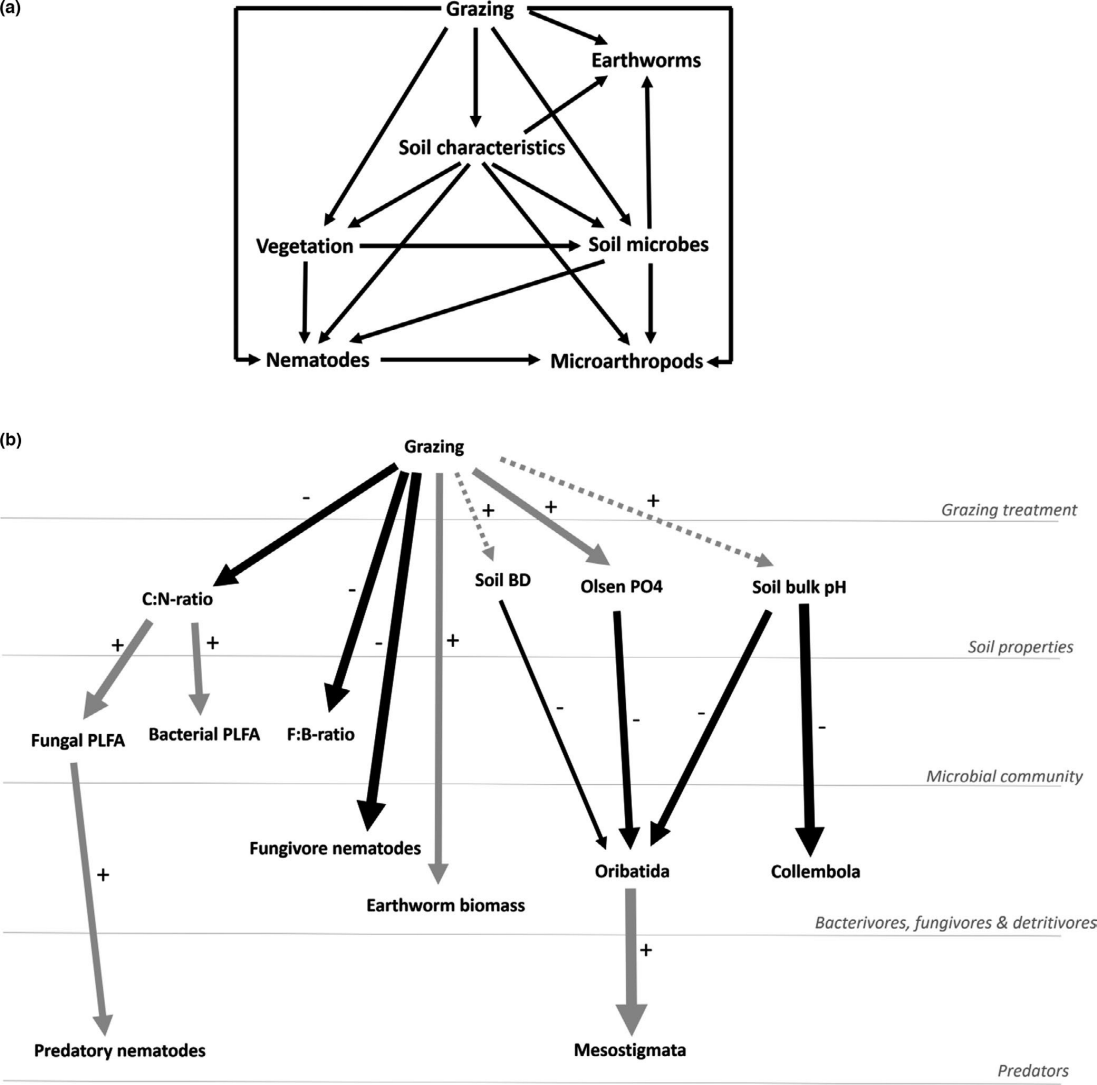
(a) Hypothesized causal relations between plant–soil ecosystem components and grazing (note: vegetation–nematode relationship was only tested for phytophagous nematodes). (b) Quantitated relationships between biotic and abiotic components of the plant–soil ecosystem from best subsets of GLMs (AICc). Arrows point in the direction of hypothesized causal relationships (− black/+ gray) with their size proportional to standardized coefficients from the optimal linear model. Dashed lines indicate nonsignificant relationships (*p* > .05) retained after AICc optimization. For visual purposes, abundance (biomass for earthworms) and not species richness is shown for invertebrates. Vegetation characteristics were not important drivers of other biotic properties and were omitted

In this study, we performed a comprehensive investigation of how long‐term grazing of birch forest patches shifted the soil physicochemical state and community structure (composition, diversity and abundance of taxonomic or functional groups) of soil organisms (microbes, nematodes, collembola, mites and earthworms). *A priori*, we hypothesized causal relationships between plant–soil ecosystem components and grazing (Figure 1a) with specific predictions concerning the different soil organisms. We predicted that the presence of cattle would increase soil nutrient content (nitrogen, phosphorus), leading to a shift from fungal to bacterial dominated soil food webs with concomitant increases and decreases in abundance of bacterivorous and fungivorous nematodes, respectively. Additionally, we predicted that grazing would have a negative effect on soil microarthropods, due to decreased pore space and increased disturbance from trampling, as observed in pastures (Schon et al., 2012). For earthworms, we predicted that faster passaging of organic matter (excreta) would benefit overall earthworm biomass, but that this would depend on differential responses of functional groups defined by their ecological niche (epigeic, surface active or anecic, deep burrowing) and vulnerability to physical disturbance of the soil surface. Finally, we expected that these processes would culminate in distinct assemblages of plants and soil organisms in the disturbed and undisturbed habitat.

## 2 MATERIALS AND METHODS

### 2.1 Birch forest ecosystem

This comparative study was carried out within the context of a long‐term natural experiment where forest patches had either been subjected to long‐term livestock grazing or left in the undisturbed seminatural state. Twenty birch (*Betula* spp.) forest patches were selected from a pool of 3161 deciduous forest patches (River Dee catchment, Aberdeenshire, Scotland) according to the presence or absence of livestock (beef cattle) grazing. Cattle densities were light to moderate (mean: 8.4 cattle ha^−1^ in 2007) and long‐term (median: 30 years) in grazed patches (Table S1.1; Figure S1.1). Ungrazed semi‐natural sites that served as the baseline comparison were ungrazed by cattle or other livestock for at least 70–100 years. All sites were, however, exposed to freely ranging wild herbivores—predominantly roe deer, *Capreolus capreolus* (Linneaus, 1758)—but their effect on the ecosystem was limited compared to the long‐term and concentrated impact of periodically confined cattle herds. Cattle grazing occurred year‐round, but with periodic and haphazard rotation of the livestock out of the woodland according to farmer judgement on the vegetation state to allow recovery. During periods of winter grazing, supplementary feed (hay) was provided at a single feeding station per site. Although we avoided sampling at these locations where cattle feeding was concentrated, this supplementary feeding represented an additional carbon input to the soil ecosystem via cattle excrement. No other systematic differences in management (e.g., forestry practices) occurred at the grazed and ungrazed sites.

### 2.2 Data collection

#### 2.2.1 Sampling design

In each site, we established a 25 m × 25 m sampling grid with 16 potential nodes at the center of the woodland (≥50 m from the forest edge). Sampling took place on randomly selected nodes of this grid (4–6 sampling points depending on the variable, with a minimum 5‐m separation) to capture within‐site spatial heterogeneity. The timing of sampling within the season was designed to coincide with the peak of activity or diversity of each taxon, while the year of sampling (2007 or 2008) was dictated by logistical constraints (see below). Because sampling points were pseudoreplicates, we pooled data for statistical analysis at the level of the forest patch site (see below).

#### 2.2.2 Forest understory plant community

The species composition and percentage (%) cover of the herbaceous understory plant community (vascular, bryophytes, and macrolichens) of each site was measured in July 2008 in a series of five quadrats (1 m^2^) randomly situated on the grid nodes. The identity and visually estimated percentage cover of each species was determined, enabling the plant species richness and percentage cover of broad plant groups (dicotyledonous herbs, graminoids, pteridophytes, and bryophytes) to be derived.

#### 2.2.3 Soil properties

In 2008, a bulked sample of six soil cores (50 mm diameter, 50 mm depth), minus the litter layer, were taken per site and analyzed for pH (measured in 0.01 M CaCl_2_ solution), phosphate levels (colorimetric PO4‐P Olsen mg/kg), and the total percentage (%) content of carbon and nitrogen using an Elementar Vario EL elemental analyzer (*n* = 20 for all). Soil bulk density (g/cm^3^) was estimated by driving a steel circular ring of standard volume (77 cm^3^) into the soil (250 mm depth) at three random grid points per site. Samples were oven dried and dry mass of soil and parent material fraction (g^−1^) obtained and converted to a volumetric mass of dry soil per sampling point. We then obtained a mean value per site of all these soil property values for subsequent analysis.

#### 2.2.4 Microbial community structure

Standard phospholipid fatty acid (PLFA) analysis was used to quantify the dry weight‐based mass of markers of fungal and bacterial biomass and the fungal‐to‐bacterial ratio in the soil samples from the sites, see Frostegård et al. (2011) for a discussion of this method. Bulked soil samples (six 25 mm diameter cores to 50 mm depth) were taken from each site in June 2008, homogenized and freeze‐dried (−20°C). We subsampled (1 g) of this freeze‐dried soil and analyzed it using GC‐MS for PLFA (µg/g) to obtain the dry weight‐based mass of markers indicating soil fungal (18:2ω6,9) and bacterial (i15:0, a15:0, 15:0, i16:0, 16:1ω7, a17:0, i17:0, cy17:0, cis18:1ω7, cy19:0) biomass. The fungal‐to‐bacterial ratio was calculated by dividing the total fungal marker by the sum of bacterial PLFA markers. The microbial community structure was characterized according to the following PLFAs. Ester‐linked branched‐chain fatty acids, indicative of Gram‐positive bacteria: i15:0, a15:0, i16:0, br17:0 and i17:0; ester‐linked monounsaturated fatty acids, such as 16:1ω5, 16:1ω7c, 18:1ω9 and 18:1ω7c and 9t; 18:1ω7c and 9t (considered mainly to be 18:1ω7 since the GC does not differentiate between these fatty acids), plus ester‐linked hydroxy fatty acids, for example, 10:03OH, 12:03OH, 14:02OH, 14:03OH, and 16:02OH. Ester‐linked polyunsaturated 18:2ω6,9 was used as an indicator of fungal biomass. Additional references used in the PLFA analysis are displayed in S2.

#### 2.2.5 Nematode abundance, richness, and trophic group

Four soil cores (25 mm diameter, depth 50 mm) were taken (May 2008) per site (80 cores in total) and weighed to determine the wet mass (g) per sample. Nematodes were extracted (48 h) using a modified Baermann funnel extraction method (van Bezooijen, 2006). The soil sample was placed into a sieve (110 mm diameter, 1 mm mesh) lined with a filter tissue (KimTech Delicate task wipe, Kimberly‐Clark™) and situated within a water‐filled funnel (150 ml) to encourage nematode migration from the soil into the water column where they gravitate into a vial (4 ml) connected to the funnel by rubber tubing.

Following extraction, we reduced the vial water volume, heated the solution (60°C) to kill the nematodes, and pipetted 2 ml of a nematode fixative solution (F.G.4:1 Formaldehyde: Glycerin + Distilled H_2_O) into each vial to preserve the specimens for counting and identification (van Bezooijen, 2006). Using a stereomicroscope (×40), we counted the number (N per 100 g sample) of nematode individuals per sample. For identification, we transferred specimens to pure glycerin via serial passaging through Seinhorst solutions (#1: 20% ethanol: 1% glycerin: 79% distilled H_2_O; #2: 95% ethanol: 5% glycerin; van Bezooijen, 2006) followed by desiccation (40°C). We pipetted and slide mounted (wax sealed) a drop of this nematode suspension from each sample. We determined the relative abundance of nematodes to the highest possible taxonomic resolution (Family/Genus) in a community subsample of 100 individuals (×1000 magnification, Leica DM12.5 microscope) and assigned them to a trophic group (e.g., predator, omnivore, bacterivore, or herbivore; Bongers, 1994). For each subsample, the number of nematodes per trophic group was divided by the dry mass (g) of the individual soil sample and multiplied by 100 to get the number of individuals per 100 g. The values for the four subsamples were then averaged to obtain a mean for each site. Observed species and their abundance are given in Table S3.1.

To describe nematode community structure, we calculated the Enrichment Index (EI), Structure Index (SI), and Channel Index (CI) using the assignment to nematode trophic groups and colonizer‐persister classes (Bongers, 1990). The EI is an indicator of soil nutrient enrichment, the SI indicates food web stability (high SI = stable food webs dominated by disturbance‐sensitive species with high longevity; Ferris et al., 2001). The CI indicates the relative importance of the fungal energy channel compared to the bacterial energy channel in the soil food web (Cesarz et al., 2015). Further description of these indices is in S4.

#### 2.2.6 Earthworms

We sampled earthworms (Lumbricidae) in May 2008 using an iron frame driven into the soil to delineate a monolith of soil (250 × 250 × 150 mm depth). These monoliths were taken from three random points on the sampling grid within each site (*n* = 60). After removing the litter layer, we excavated and sorted the soil by hand to a depth of 150 mm, earthworms collected were immediately preserved by immersion in a vessel containing 30 ml 80% Industrial Methylated Spirits solution. The species identity, ecological niche (epigeic/endogeic/anecic), total abundance, and total pooled fresh biomass (g) of earthworms per monolith sample were determined (Sims & Gerard, 1985) and are displayed in Table S3.2.

#### 2.2.7 Microarthropods

We sampled microarthropods (Collembola, Acari) with six soil cores (50 mm diameter, 50 mm depth) per site in June 2007. Microarthropods were extracted (48 h) using Tullgren extraction funnels fitted with 40 W light bulbs (Burkard Scientific Ltd). These produce a temperature gradient, exploiting the behavior of microarthropods to descend into the soil away from the light heat source, where ultimately, they fall through the funnel to be collected and preserved in a vial containing ethanol (70%). Following extraction of invertebrates, the soil was oven‐dried (105 ± 5°C, 24 h) and weighed to determine soil dry weight (g). Collembola and Acari (Oribatida, Mesostigmata) were counted and identified to species (×40 stereomicroscope, ×100 magnification Leica DM12.5 microscope) using taxonomic keys (Fjellberg, 1998; Hopkin, 2007; Krantz, 1978). Species lists are given in Table S3.3–S3.5.

### 2.3 Statistical analysis

R‐code used in this paper is accessible at Dryad (https://doi.org/10.5061/dryad.wm37pvmq2).

#### 2.3.1 Multiple impacts of grazing on the forest plant–soil ecosystem

**FIGURE 2:**
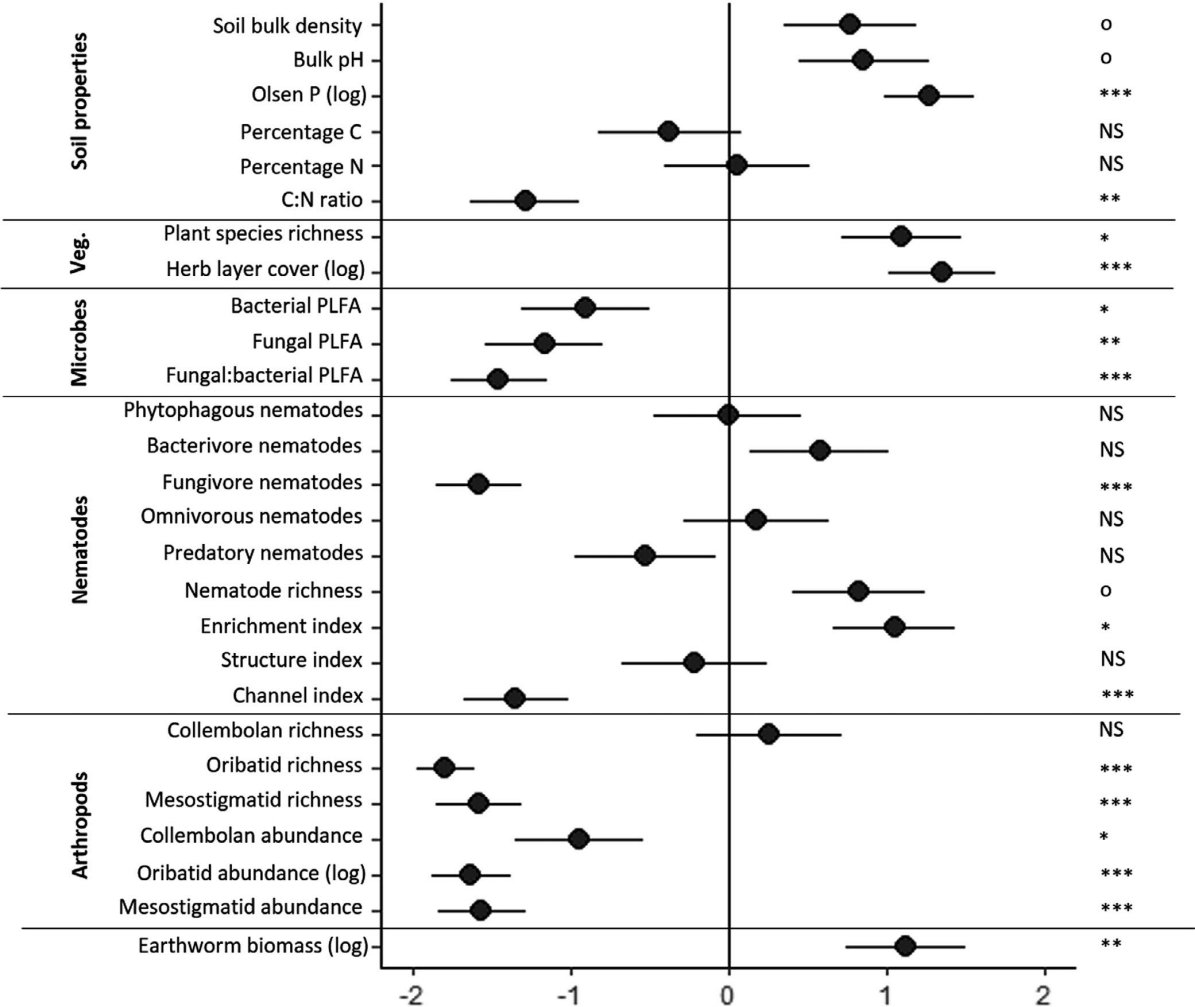
Standardized coefficients from a GLM with grazing treatment as the single explanatory variable of the response of plant–soil ecosystem components to the presence or absence of cattle grazing in forest patches (^NS^ *p* > .10; ^o^ *p *< .10; *p < .05; **p < .01; ***p < .001); Veg., Vegetation. Positive values indicate a positive correlation with presence of cattle grazing

The effect of the cattle grazing on the birch ecosystem was analyzed using general linear and best‐subset modeling (AICc for small sample sizes; Burnham & Anderson, 2002) implemented with the MuMIn package (Barton, 2020). Given the likelihood of multiple impacts of the grazing cattle, the many intercorrelated variables (Table S1.2), and the number of sites (*n* = 20), we restricted the number of candidate variables per model. Only potential causal links between the plant–soil ecosystem components and grazing according to *a priori* predictions were tested (Figure 1a). Different categories of plant–soil ecosystem variables tested were: (i) soil abiotic properties; (ii) vegetation; or communities of soil (iii) microbes, (iv) nematodes, (v) microarthropods; and (vi) earthworms. Initially, we tested the direct effect of the presence of grazing livestock (0/1) on all variables describing the ecosystem (Figure 2; Table S1.2) to assess the direct correlation. Then, we ran GLMs for each category of ecosystem variable (e.g., soil properties; vegetation; microbes; etc.). For this, we ran a two‐tier approach, first running full models for each variable category (e.g., soil model, microbial model,…) and selecting the best subset of variables by AICc optimization for each of these categories. Secondly, we compared the model of grazing presence (0/1), all the best models per category, and an intercept‐only (null) model to select the final best model based on AICc scores (Tables 1 and S5.1). Models with a ΔAICc < 2 compared to the optimal model were also considered plausible. Highly correlated explanatory variables (e.g., Figure S6.1% C & N) were not fitted to the same models. We tested for homoskedasticity (Levene) and normality of residuals (Shapiro–Wilks) and, where necessary, the response variables were log‐transformed to meet model assumptions.

**TABLE 1:** Best models for each response variable, based on AICc scores

Response variable	Expl. vars	Coeff.	SE	*t*‐value	*p*‐value
Soil properties					
Soil bulk density	Intercept	0.51	0.04	12.28	<.0001
	Grazing	0.11	0.06	1.82	.086
Soil pH	Intercept	4.90	0.13	38.73	<.0001
	Grazing	0.37	0.18	2.07	.05
log(Olsen P + 1)	Intercept	2.01	0.18	11.00	<.0001
	Grazing	1.16	0.26	4.48	.0003
C:N ratio	Intercept	18.60	0.61	30.56	<.0001
	Grazing	−3.21	0.86	−3.72	.0016
Vegetation					
Plant species richness	Intercept	−30.29	10.86	−2.79	.012
	Soil pH	9.52	2.13	4.47	.0003
log(Herb layer cover + 1)	Intercept	2.48	0.14	17.68	<.0001
	Grazing	1.01	0.2	5.07	<.0001
Microbes					
Bacterial PLFA	Intercept	−49.22	32.99	−1.49	.15
	C:N‐ratio	6.04	1.91	3.15	.006
Fungal PLFA	Intercept	−26.63	8.68	−3.07	.007
	C:N‐ratio	2.32	0.50	4.59	.0002
Fungal:bacterial PLFA	Intercept	0.26	0.010	25.83	<.0001
	Grazing	−0.069	0.014	−4.74	.0002
Nematodes					
Fungivore nematodes	Intercept	40.34	3.69	10.95	<.0001
	Grazing	−30.42	5.21	−5.84	<.0001
Predatory nematodes	Intercept	7.06	3.59	1.97	.065
	Fungal PLFA	0.70	0.24	−2.93	.009
Nematode richness	Intercept	−1.52	10.31	−0.15	.88
	Soil pH	6.40	2.02	3.16	.005
Enrichment index	Intercept	39.54	2.76	14.33	<.0001
	Grazing	10.52	3.90	2.70	.015
Channel index	Intercept	75.04	7.38	10.17	<.0001
	Grazing	−42.28	10.43	−4.05	.0007
Microarthropods					
Oribatid richness	Intercept	11.34	0.44	25.54	<.0001
	Grazing	−2.08	0.63	−3.31	.004
Mesostigmatid richness	Intercept	2.03	0.54	3.74	.002
	Orib. sp. rich	0.13	0.05	2.58	.02
Collembolan abundance	Intercept	63.78	7.33	8.70	<.0001
	Soil pH	−24.30	10.37	−2.34	.03
log(Oribatid abundance + 1)	Intercept	13.05	1.24	10.51	<.0001
	Olsen P	−0.034	0.006	−5.57	<.0001
	Soil pH	−1.37	0.25	−5.39	<.0001
	Bulk density	−2.28	0.77	−2.95	.009
Mesostigmatid abundance	Intercept	9.44	1.88	5.03	<.0001
	Oribatid ab.	0.13	0.02	8.22	<.0001
Earthworms					
log(Earthworm biomass + 1)	Intercept	0.66	0.24	2.76	.013
	Grazing	1.02	0.34	2.98	.008
Epigeic biomass	Intercept	0.36	0.25	1.45	.17
	Bacterial PLFA	0.093	0.036	2.56	.021
	Fungal PLFA	−0.022	0.011	−1.98	.065
log(Anecic biomass + 1)	Intercept	0.00	0.22	0.00	1.00
	Grazing	1.09	0.31	3.50	.003

Note

Variables that were best explained by an intercept only model (%C, %N, phytophagous nematodes, bacterivore nematodes, omnivorous nematodes, SI, collembolan richness, and endogeic earthworm biomass) are not displayed here; see Table S5.1.

#### 2.3.2 Grazing impacts on composition of plant and soil invertebrate communities

We tested how grazing affected the community composition of plants, nematodes, collembolans, oribatid mites, mesostigmatid mites, and earthworms. We used nonmetric multidimensional scaling (NMDS) to visualize differences in community composition based on a Bray–Curtis dissimilarity matrix (500 iterations; R vegan package: metaMDS function Oksanen et al., 2019). Stress scores were sufficiently low to interpret the plots in two dimensions. Data were log‐transformed to reduce the influence of the most dominant taxa. We used a Permutational Analysis of Variance (PERMANOVA, 9999 permutations; R vegan package, adonis2 function) to test if there was a significant difference in community composition between the grazed and ungrazed sites with homogeneity of variance tested using the betadisper function.

## 3 RESULTS

### 3.1 Forest soil properties

**FIGURE 3:**
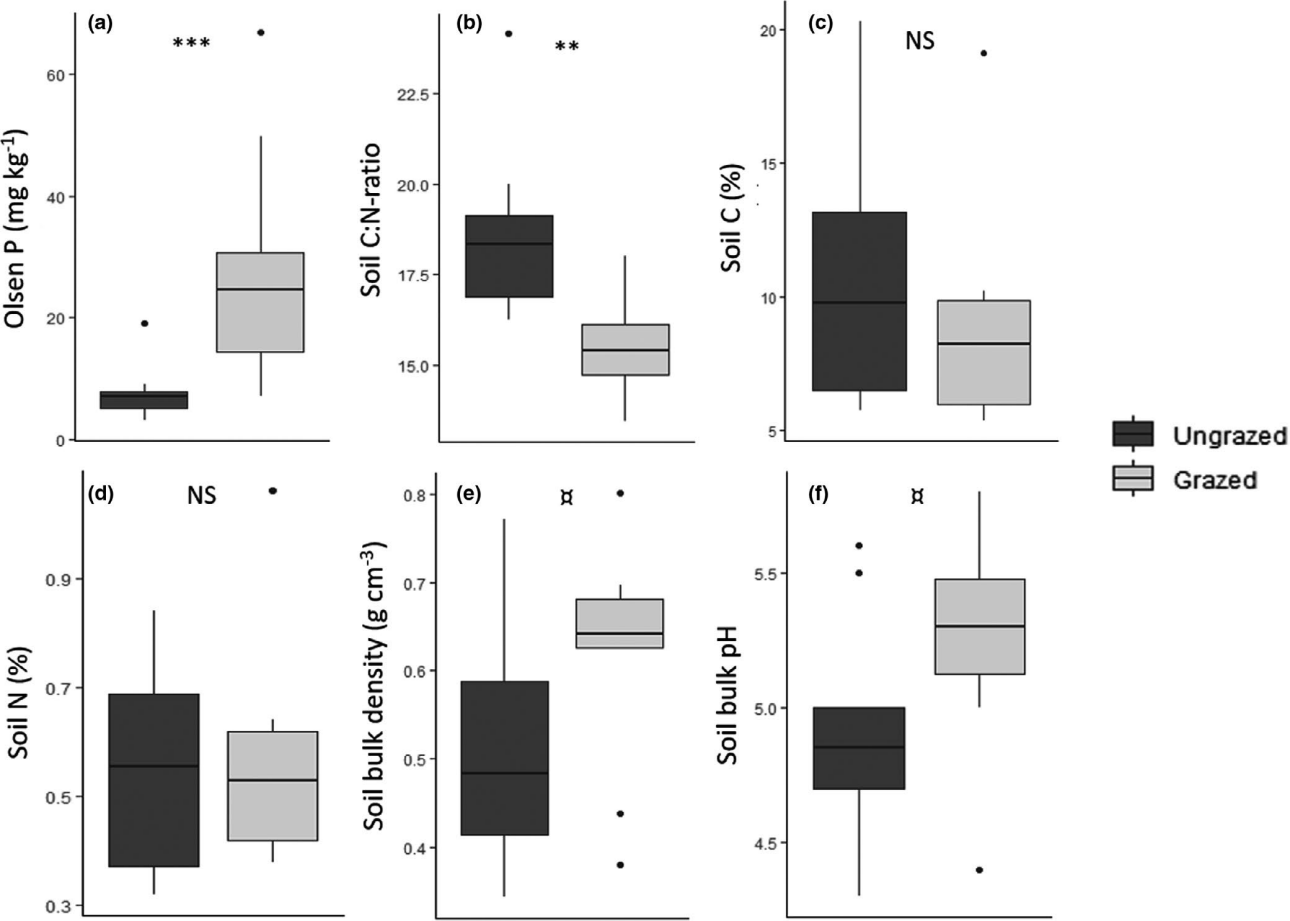
Box plots showing the effects of grazing treatment on forest soil properties. Significance levels inferred from linear models using only grazing treatment as a response variable are indicated above each figure (NS: not significant, ^¤^ *p *< .10, *p < .05, **p < .01, ***p < .001)

Cattle grazing led to a nearly four‐fold increase in available soil phosphorus levels (Table 1; Figures 1b and 3a). While no significant differences in the percentage of soil carbon and nitrogen were detected between grazed and ungrazed forests, the C:N ratio was significantly lower in grazed sites (Table 1; Figures 1b, 3b‐d and S6.1). Soil pH and bulk density were marginally (*p* < .10) higher in grazed forests, with the pH being on average 0.4 points higher in grazed patches (Table 1; Figures 1b and 3e–f), although for both variables the grazing model only marginally outperformed the intercept‐only model (ΔAICc = 1.5 and 0.6, respectively).

### 3.2 Forest understory vegetation

**FIGURE 4:**
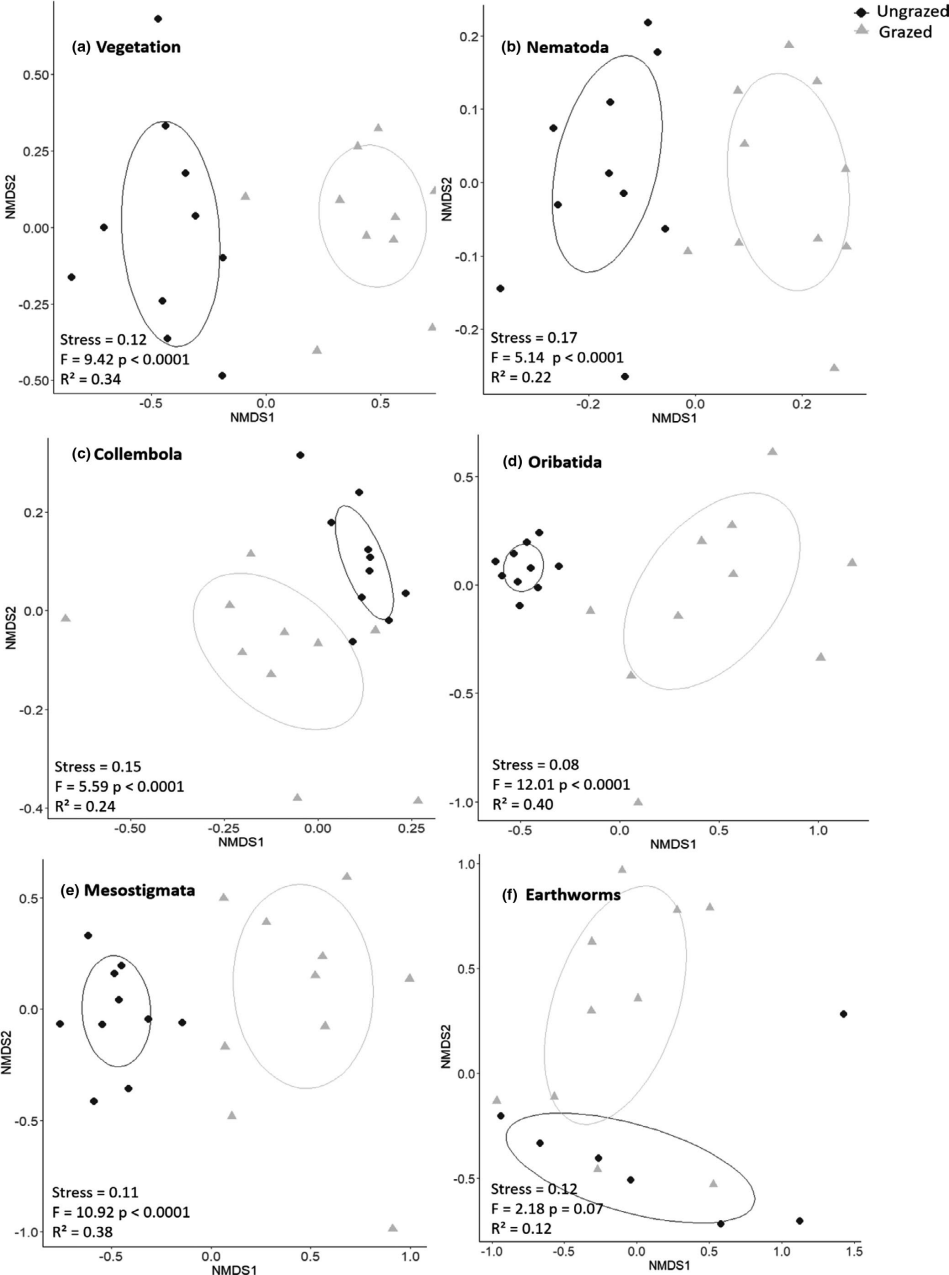
NMDS plots of (a) vegetation, (b) nematode, (c) springtail, (d) oribatid, (e) mesostigmatid, and (f) earthworm communities. Circles indicate 95% confidence range of centroid position. Stress values and PERMANOVA statistics are indicated at the lower left corner of each plot

Grazing significantly increased herb layer cover and plant species richness (Table 1; Figure 2) in the forest patches. Plant species richness was related positively (Table 1) to the marginally higher soil pH (Figures 2 and 3) in the grazed sites. Plant community composition was similarly affected by grazing with the NMDS showing a clear distinction between forest understory plant communities associated with the grazed and ungrazed habitat (Figure 4a). Changes in the vegetation were, however, not correlated with belowground changes in soil biodiversity (Table 1).

### 3.3 Soil microbial PLFA

The concentration of microbial PLFA was lower in grazed forests, with bacterial PLFA decreasing by almost a third and fungal PLFA being almost 60% lower (.2). This was correlated with the lower C:N ratio in grazed sites (Table 1; Figure 1b), which, compared to the grazing model, better explained the lower bacterial and fungal PLFA (ΔAICc = −3.9 and −6.6 respectively, Table S5.1). The stronger decline in fungal PLFA shifted the ratio of fungal to bacterial PLFA strongly toward bacteria, with bacteria representing 79.1% of all PLFA in ungrazed plots and 83.6% in grazed plots (Figure 2). The difference in fungal‐to‐bacterial PLFA ratio was best explained by the “grazing” model (Table 1).

### 3.4 Nematode trophic groups and food webs

While nematode taxonomic richness marginally increased under a grazing regime (Figure 2), this pattern was best explained by the positive correlation between soil pH and nematode richness (ΔAICc = −5.0 compared to grazing (0/1) model; Table 1). Fungivorous nematode abundance was significantly lower in grazed sites with a 75% decrease in numbers compared to the ungrazed sites (Table 1; Figures 1b and 2). Other nematode trophic groups did not show a significant response to grazing (Figure 3) with differences between sites best explained by an intercept‐only (null) model, except for predatory nematodes, which showed a positive correlation with PLFA markers of fungal biomass (ΔAICc = −5.0, compared to intercept‐only (null) mode, Tables 1 and S5.1).

Grazed forests had a significantly higher EI and a significantly lower Channel Index, while the SI did not differ significantly between grazed or ungrazed forests (Table 1; Figure 2).

### 3.5 Microarthropod abundance and richness

The presence of grazing cattle had a strong negative direct influence on the abundance and species richness of oribatid and mesostigmatid mites (Figure 2). Collembolan abundance was associated negatively with the presence of grazing, but species richness was unaffected (Table 1; Figure 2).

Oribatid abundance was best explained by a soil‐only model, containing Olsen phosphorus, soil pH and soil bulk density (Table 1). This model was considered equivalent to the grazing model (ΔAICc = −0.1; Table S5.1), which showed a negative response to grazing. Mesostigmatid abundance was better explained by a prey model containing oribatid abundance, compared to the grazing model (ΔAICc = −10.6; Tables 1 and S5.1; Figure 1b). Oribatid richness was best explained by the grazing model, which marginally outperformed a microbial model containing fungal to bacterial PLFA ratio (ΔAICc = −0.9; Tables 1 and S5.1), while for mesostigmatid richness, a prey model including oribatid richness (ΔAICc = −0.6; Tables 1 and S5.1) marginally outperformed the grazing model. Collembolan abundance was best explained by a soil model containing a negative correlation with soil pH (ΔAICc = −15.7; Tables 1 and S5.1; Figure 1b), while for collembolan richness, no model outperformed the intercept‐only model, although a soil model containing the C:N ratio and carbon content had an equal AICc‐score (ΔAICc = 0.0).

### 3.6 Earthworm biomass

**FIGURE 5:**
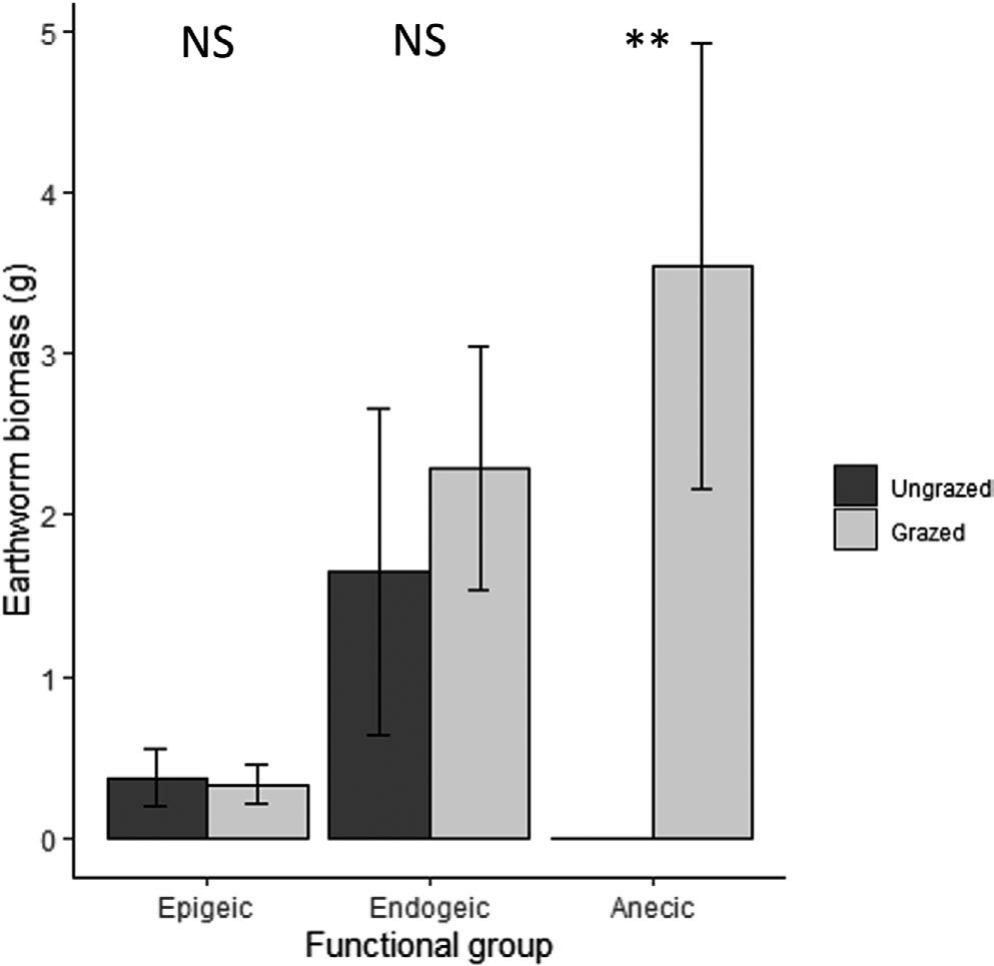
Mean (± SE) biomass of epigeic, endogeic, and anecic earthworms per habitat type (^NS^ *p* > .10; ¤*p *< .10; *p < .05; **p < .01; ***p < .001)

Total earthworm biomass was significantly higher in grazed forest patches (Table 1; Figures 1b, 3 and S6.2). However, this effect of grazing on earthworm biomass varied among functional groups. Anecic earthworms were completely absent from ungrazed plots, but the most dominant guild in grazed forests, where they represented 42.5% of earthworm biomass (Table 1; Figure 5). While grazing best explained this pattern, a soil model containing the C:N ratio was also considered (ΔAICc = −1.6). Epigeic and endogeic earthworms were unaffected by grazing (Figure 5). Epigeic earthworm biomass was positively and (marginally) negatively correlated with PLFA markers of bacterial and fungal biomass, respectively (Table 1), marginally outperforming the intercept‐only model (ΔAICc = −2.0), while endogeic biomass was best explained by the intercept‐only model, which had equal AICc scores to the soil model containing bulk pH and C:N ratio.

### 3.7 Soil invertebrate assemblage composition

NMDS and PERMANOVA analysis revealed clearly distinct assemblages of nematodes, collembolans, oribatids, mesostigmatids, and earthworms associated with the grazed or ungrazed forest patches (Figure 4b–f). While the oribatid assemblages showed a much lower dispersion in ungrazed plots (*p* = .0001), separation between the two groups was sufficiently strong to consider the results reliable, given the robustness of this test for balanced designs (Anderson & Walsh, 2013).

## 4 DISCUSSION

Long‐term cattle grazing consistently transformed the plant–soil assemblages in these replicated forest patches by modifying the abiotic soil environment and directly—or indirectly via changes in the soil physicochemical state or biological associations—the abundance, diversity, and composition of soil biota. One major functional consequence of the long‐term introduction of cattle to this ecosystem was the reduction of the importance of the fungal energy channel, lower microarthropod abundance and diversity, and a functional shift in earthworm community composition toward anecic (deep burrowing) species dominance.

Through their digestion of plant material and deposition as manure, grazing of cattle in this ecosystem of forest patches elevated soil phosphate levels, decreased the soil C:N‐ratio, and marginally increased soil pH. The reduced carbon loading of the soil ecosystem (i.e., observed decrease in C:N ratio) can be explained by the rapid (c.f. slower fungal decomposition cycling) conversion of vegetation to manure and external inputs from supplementary feeding (hay) of the livestock. A nonmutually exclusive explanation could also be the indirect effects of grazing on the forest understory vegetation. As previously shown for this ecosystem (Vanbergen et al., 2006, 2014), plant community structure was greatly modified by cattle presence with percentage cover of herbs and plant species richness significantly higher in grazed forest patches. These compositional changes in the forest understory vegetation were, however, uncorrelated with specific parameters of soil state (apart from a positive correlation between higher plant richness and soil pH) or biotic community structure. A potential, but unmeasured, plant‐mediated explanation to the observed changes in soil nutrient content may come from grazing‐induced changes to plant NPP (Hao & He, 2019; Wardle & Bardgett, 2004), root exudation, or chemical quality of tissues and hence litter inputs (Bardgett & Wardle, 2003; Grayston et al., 1996; Hamilton et al., 2008).

The soil physical structure was similarly modified in the grazed habitat with slightly elevated soil bulk density implying compaction from trampling by herds, which reduces soil porosity and water retention capacity (Sharrow, 2007).

Carbon is the limiting nutrient for microbial detritivores outside the rhizosphere (Grayston et al., 1996; Scheu et al., 2005). Here, cattle grazing reduced the overall soil microbial biomass, which corresponded with the fall in the C:N ratio. Similar declines in microbial biomass have been reported in European submontane grassland pastures under intensive grazing (Bardgett et al., 2001), although in subtropical pastures, grazing can increase microbial biomass (Wang et al., 2006), pointing to how environmental zone can introduce context dependencies. Additionally, since microbial biomass might respond in a nonlinear way, a gradient in grazing intensity might reveal patterns obscured by our binary approach (Bardgett et al., 2001). Although N‐saturated soils may show declines in soil microbes (Wallenstein et al., 2006), we found no evidence that grazing shifted the soil N level.

Fungal biomass was more negatively affected than the bacterial component, potentially because fungi are more associated with soils with high C:N ratios (Oates et al., 2012; Wan et al., 2015), recalcitrant litter (Högberg et al., 2007), and mycelia are more susceptible to damage from physical disturbances, that is, trampling and soil compaction (Hartmann et al., 2014). The shift in the dominance structure of the soil microbial community, here caused by a reduction in fungal biomass in the grazed sites, is consistent with previous studies that show grazing‐induced shifts toward the bacterial energy channel (Lopez‐Sangil et al., 2011; Oates et al., 2012; Waring et al., 2013). Shifts from fungal to bacterial decomposition have been associated with changes in carbon sequestration (Malik et al., 2016), but the magnitude of the effect in this study system was insufficient to alter soil carbon content.

This shift to more bacteria‐dominated soil food webs in grazed forest patches mediated through changes in nutrient ratios led to corresponding knock‐on effects on the soil nematode assemblage. Fungivorous taxa were the only nematode trophic group to be significantly less abundant in grazed forest patches, but a significant positive correlation between fungal biomass and the presence of predatory nematodes suggests a possible bottom‐up conduit in the nematode food web connecting fungivorous nematodes and their predators (Wardle & Yeates, 1993). The significantly lower nematode Channel Index in grazed forest fragments indicates a shift from fungivorous to bacterivorous nematode dominance. This provides further evidence of a decreased energy flow through the fungal channel in the grazed forest patches. This is in line with studies on fertilized grasslands (Parfitt et al., 2010) and links to relatively higher soil N content (Cesarz et al., 2015), but contrasts with other studies that found a positive correlation, or no correlation at all between grazing and the Channel Index in grassland ecosystems (Briar et al., 2012; Mills & Adl, 2011). This is likely due to the fact that the bacterial energy channel usually predominates in intensively grazed grasslands, while forest soils generally have a dominant fungal energy channel (Ruess, 2003). While different trophic groups contrasted in their responses to grazing and linked environmental characteristics, total nematode richness was positively correlated with relatively greater soil pH in grazed habitat (>5.0) and consistent with the optimal pH range (5.0–7.0) for most nematode genera (Mulder et al., 2003; Nisa et al., 2021).

This shift in the dominance of nematode groups was also reflected in a grazing‐associated increase in the EI, caused by faster nutrient cycling through input of more labile organic matter and a proliferation of more opportunistic bacterial‐feeding nematodes (Ferris et al., 2001). This response of the nematode assemblage structure to enrichment can be idiosyncratic with positive and negative responses seen in grassland systems often dependent on grassland habitat type and grazing intensity (Hu et al., 2015; Wang et al., 2006, 2018).

The observed nutrient‐driven shifts in nematode assemblages to a bacterial energy channel did not translate, however, into an overall reduction in the stability or complexity of the nematode food web (SI; Ferris et al., 2001). Trophic linkages were rarely detected in our models. This may reflect the spatial scale at which we undertook our analysis, with effects attributable to grazing detectable, but lacking the finer spatio‐temporal resolution to detect the signal of soil species interactions and their cascading effects across the soil food web (Thakur et al., 2020). Consequently, subtler trophic interactions went undetected and only the modified nutrient cycling that shifted the relative dominance of nematode feeding groups in the micro‐food web was observed.

Microarthropod abundance and species richness (excepting Collembola) were strongly reduced by grazing in these forest patches, which was linked to changes in the soil physicochemical state (elevated phosphorous content, bulk density and pH). By inhabiting the interstitial soil pore space, microarthropods may have suffered greater direct mortality from soil compaction by cattle trampling as well as loss of microniche space or nutritional resources with the physical disturbance of the litter layer (Bardgett et al., 1998; Hopkin, 1997; Wardle et al., 2001). Larger bodied arthropods are more sensitive to the adverse effects of grazing (and other disturbances) than small‐bodied species (Wardle & Bardgett, 2004). Here, due to their small size nematodes were unaffected by soil compaction (Bouwman & Arts, 2000; Schon et al., 2012), while mites showed a far stronger negative response than collembolans (Andriuzzi & Wall, 2017). In particular, oribatid mites are known to be associated with acidic soils (Maraun & Scheu, 2000) and are very sensitive to physical disturbances (Schon et al., 2012) with low resilience due to longer generation times and “K‐selected” traits (Cole et al., 2008). Collembolans were also more abundant on the more acidic soils of the ungrazed habitat, possibly due to physiological adaptation to that niche or their role as grazers of the fungi typically associated with more acidic soils such as under forest (Hopkin, 1997; Vanbergen et al., 2007), although we found no direct correlation between collembolan abundance and PFLA markers of fungal biomass here.

Grazing also produced distinct microarthropod assemblages. Mites, particularly oribatids, and to a lesser extent collembolans, showed a much higher dispersion of community composition in grazed sites compared to the relatively homogenous communities in ungrazed forest patches. This suggests that cattle grazing in these forest patches created a higher level of beta‐diversity between sites. The mechanism behind this remains to be established. However, it would be consistent with greater soil microhabitat or niche diversity arising from the actions of cattle on the plant–soil ecosystem.

As predicted, the response of earthworms to grazing was contingent on their exact ecological role and function. Epigeic and endogeic earthworm biomass did not differ significantly between grazed and ungrazed sites, this was unexpected for epigeic species that are vulnerable to the effects of trampling (Schon et al., 2011). Manure can be a food resource for earthworms (Bacher et al., 2018; Curry et al., 2008) and hence the addition of this partly digested organic matter input to these forest soils may have compensated for adverse effects associated with any physical disturbance by the grazing livestock. Strikingly, anecic earthworms were absent from ungrazed sites, whereas they were the most dominant earthworm functional group in grazed forest patches. Because of their vertical burrowing behavior, anecic earthworms can benefit from manure input at the soil surface, while their deep vertical burrows protect them from trampling by cattle (Schon et al., 2012). This may explain the observed increase in anecic earthworm biomass associated with grazing, as reported from grassland systems (Muldowney et al., 2003; Schon et al., 2011). This has implications for ecosystem functioning as anecic earthworms vertically transport nutrients and organic material (Don et al., 2008) and increase nitrogen mineralization (Van Groenigen et al., 2014). Although they may compensate for soil compaction caused by cattle (Capowiez et al., 2012) and form more stable microhabitats for soil arthropods, rich in nutrients and micro‐organisms (Eisenhauer, 2010), in this system, their uplift in biomass was insufficient to offset the other impacts.

Overall, habitat disturbance of birch forest patches by long‐term grazing by ungulates profoundly modified the plant–soil subsystem probably via the combination of trampling, biomass consumption, and excreta. Specific shifts in nematode community trophic structure indicated a reduction in the predominance of energy flow through the fungal pathway and a subtle shift toward a soil food web underpinned by bacterial decomposition of more labile organic matter. Such results demonstrate the ecosystem engineering potential of large ungulate grazers in forest systems, however, the consequences for biodiversity–ecosystem function relationships (e.g., nutrient cycles, soil–atmosphere gas exchanges) and species interactions remains to be understood.

## CONFLICT OF INTEREST

The authors declare no conflict of interest.

## AUTHOR CONTRIBUTION

**Willem Proesmans:** Formal analysis (lead); Visualization (lead); Writing – original draft (lead). **Christopher Andrews:** Investigation (equal); Writing – review & editing (supporting). **Alan Gray:** Investigation (equal); Writing – review & editing (supporting). **Rob Griffiths:** Investigation (equal); Writing – review & editing (supporting). **Aidan Keith:** Investigation (equal); Writing – review & editing (supporting). **Uffe N. Nielsen:** Investigation (equal); Writing – review & editing (supporting). **David Spurgeon:** Investigation (equal); Writing – review & editing (supporting). **Richard Pywell:** Conceptualization (equal); Funding acquisition (equal); Project administration (equal); Writing – review & editing (supporting). **Bridget Emmett:** Conceptualization (equal); Funding acquisition (equal); Project administration (equal); Writing – review & editing (supporting). **Adam J. Vanbergen:** Conceptualization (equal); Funding acquisition (equal); Investigation (equal); Project administration (equal); Supervision (lead); Writing – review & editing (lead).

## Supporting information

Supinfo S1

## Data Availability

Raw ecological data and R‐code: Dryad https://doi.org/10.5061/dryad.wm37pvmq2.
