#  

**DOI:** 10.1002/ece3.8873

**Published:** 2022-04-28

In the recent article by Proesmans et al. (2022), the first affiliation should read “Agroécologie, INRAE, Institut Agro, Univ. Bourgogne, Univ. Bourgogne Franche‐Comté, F‐21000 Dijon, France.”

The authors apologize for the error.
