# Supplementary material for: Long‐term cattle grazing shifts the ecological state of forest soils
Source: Ecol Evol. 2022 Mar 31;12(4):e8786. doi: 10.1002/ece3.8786 (PMC8969921; doi:10.1002/ece3.8786)
Supplement: Supplementary file 1 — Supinfo S1 [file ECE3-12-e8786-s001.docx]

Supplement S1: Site description and environmental variables

*Table S1.1: Name, location, area and management of the 20 selected birch forest fragments in Aberdeenshire, Scotland. Soil types were determined from soil maps (Ordnance Survey: Soil Survey of Scotland 1959, The Macaulay Institute). Management information was collected by questionnaires sent to farmers - no data means no response was forthcoming.*

|  | **Birch wood site** | **Latitude** | **Longitude** | **Area** | **Altitude** | **Soil type** | **Cattle ha^-1^** | | **Years grazed** |
| --- | --- | --- | --- | --- | --- | --- | --- | --- | --- |
|  |  | **Decimal degrees** | | **(ha)** | **(m.a.s.l)** |  | **2003** | **2007** |  |
| 1 | Allancreich | 57.0601 | -2.7029 | 2.93 | 150 | Brown forest | 25.17 | 25.17 | >30 |
| 2 | Balnagowan farm | 57.0909 | -2.7992 | 22.09 | 150 | Iron podzol | 2.00 | No data | >30 |
| 3 | Balnagowan hill | 57.0956 | -2.8056 | 4.62 | 150 | Iron podzol | 3.20 | No data | >30 |
| 4 | Beechgrove | 57.0367 | -2.5124 | 2.28 | 100 | Gley | 1.50 | 8.42 | 35 |
| 5 | Bridgend | 57.1123 | -2.8209 | 8.39 | 140 | Iron podzol | 0.00 | 9.95 | 40 |
| 6 | Corntulloch | 57.0642 | -2.9204 | 11.05 | 180 | Brown forest | 5.15 | 4.00 | 30 |
| 7 | Dinnet | 57.0804 | -2.8935 | 24.88 | 160 | Brown forest | 3.69 | 6.33 | >20 |
| 8 | Greystone | 57.0532 | -2.9372 | 0.37 | 220 | Brown forest | 0.00 | 4.07 | 70 |
| 9 | Netherton | 57.0689 | -2.8958 | 3.19 | 190 | Brown forest | 1.28 | 0.92 | 25 |
| 10 | Potarch | 57.0660 | -2.6544 | 0.58 | 100 | Brown forest | No data | 0.00 | 20 |
| 11 | Airfield | 57.0783 | -2.8282 | 18.87 | 150 | Brown forest | Ungrazed | Ungrazed | 0 |
| 12 | Backhill | 57.0644 | -2.5799 | 47.08 | 80 | Brown forest | Ungrazed | Ungrazed | 0 |
| 13 | Braehead | 57.0581 | -2.9621 | 1.51 | 190 | Brown forest | Ungrazed | Ungrazed | 0 |
| 14 | Brathens | 57.0741 | -2.5321 | 28.44 | 125 | Iron podzol | Ungrazed | Ungrazed | 0 |
| 15 | Dalhaikie | 57.0756 | -2.5996 | 22.49 | 145 | Brown forest | Ungrazed | Ungrazed | 0 |
| 16 | Easter Sluie | 57.0617 | -2.6134 | 11.57 | 110 | Brown forest | Ungrazed | Ungrazed | 0 |
| 17 | St. James | 57.0800 | -2.8818 | 1.58 | 160 | Brown forest | Ungrazed | Ungrazed | 0 |
| 18 | Scolty hill | 57.0430 | -2.5141 | 1.75 | 105 | Brown forest | Ungrazed | Ungrazed | 0 |
| 19 | Sunnyview | 57.0696 | -2.5879 | 1.60 | 95 | Brown forest | Ungrazed | Ungrazed | 0 |
| 20 | Woodfield | 57.1353 | -2.7988 | 12.47 | 230 | Iron podzol | Ungrazed | Ungrazed | 0 |


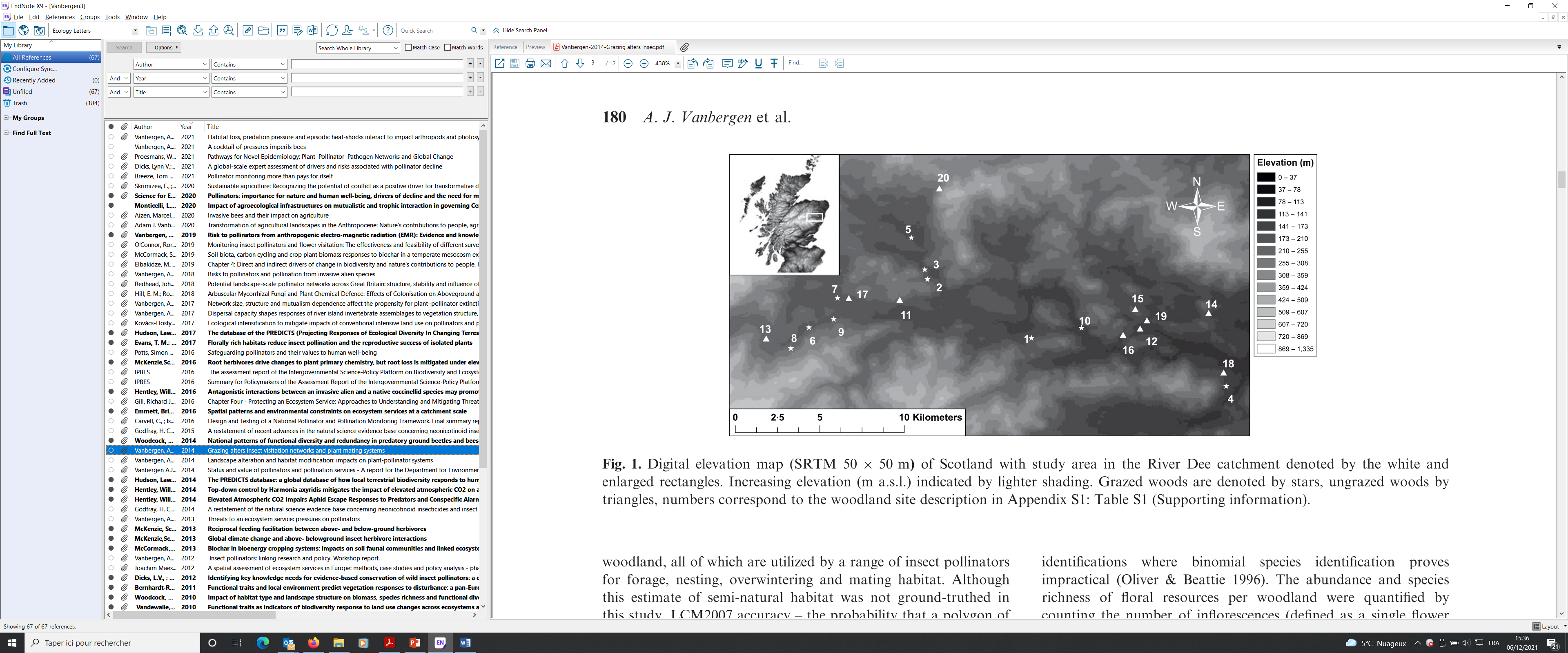


*Fig. S1.1 Digital elevation map (SRTM 50 9 50 m) of Scotland with study area in the River Dee catchment denoted by the white and enlarged rectangles. Increasing elevation (m) indicated by lighter shading. Grazed woods are denoted by stars, ungrazed woods by triangles, numbers correspond to the woodland site description in Appendix S1: Table S1.*

Table S1.2 : Variables used in the study per treatment category. Range indicates minimal, mean and maximal value for each variable per treatment.

| **Category** | **Variable** | **Range (Ungrazed)** | **Range (grazed)** |
| --- | --- | --- | --- |
| Treatment | Grazing | Ungrazed (N = 10) | Grazed (N = 10) |
| Soil characteristics | Soil Bulk density (g cm^-3^) | 0.34 – 0.51 – 0.77 | 0.38 – 0.62 – 0.80 |
|  | Carbon content (%) | 5.7 – 10.5 – 20.3 | 5.4 – 8.9 – 19.1 |
|  | Nitrogen content (%) | 0.32 – 0.55 – 0.84 | 0.38 – 0.56 – 1.06 |
|  | C:N-ratio | 16.3 – 18.6 – 24.2 | 13.4 – 15.42 – 18.02 |
|  | Olsen P (mg kg^-1^) | 3.0 – 7.3 – 19.0 | 7.0 – 27.7 – 67.0 |
|  | Soil pH | 4.3 – 4.9 – 5.6 | 4.4 – 5.3 – 5.8 |
| Vegetation | Herb layer cover (%) | 8.0 – 11.4 – 21.0 | 10.8 – 36.2 – 74.6 |
|  | Plant species richness | 10 – 15 – 20 | 13.0 – 21.2 – 30.0 |
| Microbes | Bacterial PLFA (µg g^-1^) | 26.3 – 65.0 – 108.8 | 22.2 – 42.1 – 93.5 |
|  | Fungal PLFA (µg g^-1^) | 5.8 – 17.4 – 31.8 | 4.9 – 8.2 – 18.7 |
|  | Fungal :bacterial PLFA | 0.21 – 0.26 – 0.34 | 0.16 – 0.20 – 0.23 |
| Nematodes | Plant associated nematodes ab. | 17.2 – 42.2 – 70.2 | 14.1 – 42.1 – 77.0 |
|  | Bacterivore nematodes ab. | 26.0 – 50.8 – 83.3 | 42.8 – 63.2 – 101.6 |
|  | Fungivore nematodes ab. | 10.7 – 40.3 – 57.4 | 1.6 – 9.9 – 26.9 |
|  | Predatory nematodes ab. | 3.7 – 18.6 – 37.2 | 7.1 – 13.5 – 31.6 |
|  | Omnivorous nematodes ab. | 3.8 – 18.4 – 41.9 | 0.55 – 20.6 – 46.5 |
|  | Nematode richness | 25.0 – 29.1 – 34.0 | 23.0 – 32.9 – 40.0 |
|  | Enrichment Index (EI) | 32.0 – 39.5 – 44.0 | 33.2 – 50.1 – 70.4 |
|  | Structure Index (SI) | 59.5 – 75.1 – 87.1 | 59.1 – 73.1 – 83.5 |
|  | Channel Index (CI) | 31.1 – 75.0 – 100.0 | 6.6 – 32.8 – 79.8 |
| Microarthropods | Oribatid richness | 25.0 – 29.6 – 38.0 | 7.0 – 13.1 – 17 |
|  | Oribatid abundance | 73.8 – 158.5 – 232.0 | 11.2 – 33.5 – 82.0 |
|  | Mesostigmatid richness | 11.0 – 12.2 – 15.0 | 3.0 – 7.1 – 12.0 |
|  | Mestostigmatid abundance | 17.7 – 30.1 – 40.3 | 5.3 – 13.1 – 20.7 |
|  | Collembolan richness | 9.0 – 10.3 – 12.0 | 8.0 – 10.6 – 12.0 |
|  | Collembolan abundance | 25.5 – 63.8 – 86.3 | 11.8 – 39.5 – 109.2 |
| Earthworms | Earthworm biomass (g) | 0.0 – 2.1 – 14.5 | 1.3 – 5.7 – 17.5 |
|  | Epigeic biomass (g) | 0.0 – 0.4 – 1.6 | 0.0 – 0.3 – 1.0 |
|  | Endogeic biomass (g) | 0.0 – 1.6 – 10.2 | 0.0 – 2.3 – 7.1 |
|  | Anecic biomass (g) | 0.0 – 0.0 – 0.0 | 0.0 – 3.5 – 12.1 |

Supplement S2: References for PLFA-analysis

Bååth, E., & Anderson, T. H. (2003). Comparison of soil fungal/bacterial ratios in a pH gradient using physiological and PLFA-based techniques. *Soil Biology and Biochemistry*, *35*(7), 955-963.

Galbraith, L., & Wilkinson, S. G. (1991). Polar lipids and fatty acids of Pseudomonas caryophylli, Pseudomonas gladioli and Pseudomonas pickettii. *Microbiology*, *137*(1), 197-202.

Haack, S. K., Garchow, H., Odelson, D. A., Forney, L. J., & Klug, M. J. (1994). Accuracy, reproducibility, and interpretation of fatty acid methyl ester profiles of model bacterial communities. *Applied and Environmental Microbiology*, *60*(7), 2483-2493.

O’Leary W. M. and Wilkinson S. G. (1988) Gram-positive bacteria. In Microbial Lipids (C. Ratledge and S. G. Wilkinson, Eds), Vol. 1, pp. 117-201. Academic Press, London.

Petersen, Søren O., and Michael J. Klug. "Effects of sieving, storage, and incubation temperature on the phospholipid fatty acid profile of a soil microbial community." *Applied and environmental microbiology* 60.7 (1994): 2421-2430.

Ratledge, Colin, and Stephen G. Wilkinson, eds. *Microbial lipids*. Vol. 2. Academic press, 1988.

Zelles L., Rackwitz R., Bai Q.Y., Beck T., Beese F. (1995) Discrimination of microbial diversity by fatty acid profiles of phospholipids and lipopolysaccharides in differently cultivated soils. In: Collins H.P., Robertson G.P., Klug M.J. (eds) The Significance and Regulation of Soil Biodiversity. Developments in Plant and Soil Sciences, vol 63. Springer, Dordrecht. https://doi.org/10.1007/978-94-011-0479-1_9

Supplement S3: Species lists

Table S3.1: Nematode taxa found in this study, including c-p score, trophic guild and total abundance. Ba = bacterivore, Fu = Fungivore, Om = Omnivore, Pl = Plant-associated/herbivore, Pr = Predatory.

| **Taxon** | **c-p score** | **Trophic** | **Total abundance** |
| --- | --- | --- | --- |
| *Acrobeloides* | 2 | Ba | 541 |
| *Aglenchus* | 2 | Pl | 2 |
| *Alaimus* | 4 | Ba | 32 |
| *Amphidelus* | 4 | Ba | 3 |
| *Anaplectus* | 2 | Ba | 225 |
| *Aphanolaimus* | 3 | Ba | 13 |
| *Aphelenchoides* | 2 | Fu | 877 |
| *Aphelenchus* | 2 | Fu | 32 |
| *Aporcelaimellus* | 5 | Om | 350 |
| *Aporcelaimus* | 5 | Om | 47 |
| *Bastiana* | 3 | Ba | 1 |
| *Bitylenchus* | 3 | Pl | 12 |
| *Boleodorus* | 2 | Pl | 19 |
| *Bunonema* | 1 | Ba | 4 |
| *Cephalobus* | 2 | Ba | 35 |
| *Chiloplacus* | 2 | Ba | 7 |
| *Chiloplectus* | 2 | Ba | 25 |
| Chromadorid X | 3 | Om | 7 |
| *Clarkus* | 3 | Ba | 209 |
| *Coomansus* | 4 | Pr | 12 |
| *Coslenchus* | 2 | Pl | 337 |
| *Diptherophora* | 3 | Fu | 7 |
| *Ditylenchus* | 2 | Fu | 19 |
| *Dorydorella* | 4 | Om | 2 |
| *Dorylaimellus* | 5 | Pl | 11 |
| *Enchodelus* | 4 | Pr | 15 |
| *Epidorylaimus* | 4 | Om | 170 |
| *Eucephalobus* | 2 | Ba | 479 |
| *Eudorylaimus* | 4 | Om | 688 |
| *Eumonhystera* | 2 | Ba | 108 |
| *Filenchus* | 2 | Fu | 1129 |
| *Geomonhystera* | 2 | Ba | 1 |
| *Helicotylenchus* | 3 | Pl | 318 |
| *Hemicycliophora* | 3 | Pl | 11 |
| *Heterocephalobus* | 2 | Ba | 95 |
| *Ironus* | 4 | Pr | 1 |
| *Malenchus* | 2 | Pl | 17 |
| *Mesodorylaimus* | 4 | Om | 453 |
| *Metateratocephalus* | 3 | Ba | 85 |
| *Microdorylaimus* | 4 | Om | 25 |
| *Mononchus* | 4 | Pr | 9 |
| *Mylonchulus* | 4 | Pr | 17 |
| *Nagelus* | 3 | Pl | 1 |
| *Nygolaimus* | 5 | Pr | 1 |
| *Panagrolaimus* | 1 | Ba | 285 |
| *Paratylenchus* | 2 | Pl | 752 |
| *Plectus* | 2 | Ba | 1481 |
| *Pratylenchus* | 3 | Pl | 298 |
| *Prionchulus* | 4 | Pr | 305 |
| *Prismatolaimus* | 3 | Ba | 99 |
| *Pristionchus* | 1 | Pr | 6 |
| *Prodesmodora* | 3 | Ba | 91 |
| *Prodorylaimus* | 4 | Om | 34 |
| *Pungentus* | 4 | Om | 108 |
| *Rhabditidae* | 1 | Ba | 272 |
| *Rhabdolaimus* | 3 | Ba | 15 |
| *Rotylenchus* | 3 | Pl | 9 |
| *Scutylenchus* | 3 | Pl | 13 |
| *Stenonchulus* | 3 | Pr | 9 |
| *Teratocephalus* | 3 | Ba | 87 |
| *Thonus* | 4 | Om | 24 |
| *Tripyla* | 3 | Pr | 156 |
| *Tylencholaimus* | 4 | Fu | 921 |
| *Tylenchorhynchus* | 3 | Pl | 101 |
| *Tylenchus-type* | 2 | Pl | 15 |
| *Tylolaimophorus* | 3 | Fu | 97 |
| *Wilsonema* | 2 | Ba | 12 |

Table S3.2 : Earthworms encountered in the study and their ecology

| **Species** | **Ecology** |
| --- | --- |
| *Aporrectodea caliginosa caliginosa* | Endogeic |
| *Aporrectodea caliginosa nocturna* | Anecic |
| *Aporrectodea caliginosa tuberculata* | Endogeic |
| *Allolobophora chlorotica* | Endogeic |
| *Aporrectodea longa* | Anecic |
| *Aporrectodea rosea* | Endogeic |
| *Lumbricus castaneus* | Epigeic |
| *Lumbricus rubellus* | Epigeic |
| *Lumbricus terrestris* | Anecic |
| *Octolasion cyaneum cyaneum* | Endogeic |

Table S3.3 : Collembolan taxa and their total abundance

| **Taxon** | **Total abundance** |
| --- | --- |
| *Anurida granaria* | 58 |
| *Ceratophysella denticulata* | 151 |
| *Folsomia quadrioculata* | 1481 |
| *Freisea* sp | 101 |
| *Isotomiella minor* | 726 |
| *Isotomurus palustris* | 392 |
| *Lepidocyrtus lignorum* | 166 |
| *Mesaphorura* sp | 1203 |
| *Paratullbergia callipygos* | 289 |
| *Parisotoma notabilis* | 1067 |
| *Protophorura armata* group | 298 |
| *Sphaeridia pumilis* | 87 |

Table S3.4 : Oribatid mite taxa and their total abundance

| **Site** | **Total abundance** |
| --- | --- |
| *Autogneta* sp1 | 91 |
| *Autogneta* sp2 | 16 |
| *Banksinoma lanceolata* | 99 |
| *Berniella* sp. | 35 |
| *Carabodes femoralis* | 1 |
| *Carabodes labyrinthicus* | 8 |
| *Carabodes willmanni* | 2 |
| *Cepheus* sp1 | 10 |
| *Ceratoppia bipilis* | 8 |
| *Ceratozetes gracilis* | 324 |
| *Chamobates cuspidatus* | 172 |
| *Damaeus (Adamaeus) onustus* | 8 |
| *Damaeus (Damaeus) riparius* | 1 |
| Damaiedae, *Porobelba* (?) | 12 |
| *Dameobelba minutissima* | 7 |
| *Dissorhina ornata* | 43 |
| *Edwardzetes edwardsi* | 8 |
| *Eulohmannia ribagai* | 5 |
| *Eupelops plicatus* | 73 |
| *Eupelops torulosus* | 3 |
| *Euzetes nitens* | 31 |
| *Hypochthonius rufulus* | 5 |
| *Inscultoppia* sp. | 6 |
| *Lauroppia* sp. | 61 |
| *Leibstadia similis* | 113 |
| *Malaconothrus monodactylus* | 18 |
| *Medioppia subpectinata* | 754 |
| *Microppia minus* | 50 |
| *Minunthozetes* | 91 |
| *Nanhermannia coronota* | 248 |
| New sp 3 (S87) | 1 |
| New sp 4 (S88) | 1 |
| New sp 5 (S117) | 1 |
| New sp 6 (S119) | 1 |
| New sp1 (S20) | 156 |
| New sp2 (S6) | 4 |
| *Nothrus palustris* | 11 |
| *Nothrus silvestris* | 111 |
| *Odontocepheus elongatus* | 3 |
| *Ophidiatrichus tecta* | 36 |
| *Oppiella nova* | 3415 |
| *Oribatula venusta* | 12 |
| *Parachipteria punctata* | 79 |
| *Pergalumna* sp1 | 5 |
| *Phthiracaridae* sp | 104 |
| *Phthiracarus* sp1 | 218 |
| *Phthiracarus* sp2 | 2 |
| *Platynothrus peltifer* | 105 |
| *Quadroppia quadricarinata* | 223 |
| *Rhysotritia duplicata* | 25 |
| *Scheloribates* (?) | 1 |
| *Steganacarus magnus* | 323 |
| *Suctobelbella* sp 1 | 17 |
| *Suctobelbella* sp 2 | 6 |
| *Suctobelbella* sp 3 | 37 |
| *Suctobelbella* sp 4 | 5 |
| *Suctobelbella* sp 5 | 1144 |
| *Tectocepheus minor* | 230 |
| *Trichobates* (?) | 5 |
| *Tritegeus bisulcatus* | 3 |

Table S3.5 : Mesostigmatid mite taxa encountered in the study and their total abundance.

| **Taxon** | **Total abundance** |
| --- | --- |
| *Epicrius* sp. 1 | 2 |
| Eviphididae, *Eviphis ostrinus* (?) | 46 |
| *Geholaspis longispinosus* | 4 |
| *Geholaspis mandibularis* | 17 |
| Holoparisitus, *Schizosthetus* (?) | 6 |
| Macrochelidae: *Macrocheles* sp. 1 | 8 |
| Pachylaelaphidae, *Onchodelus* (?) | 16 |
| Parasitinae, sp 1 | 8 |
| Parasitinae, sp 2 | 3 |
| *Pergamasus integer* | 104 |
| *Pergamasus parrunciger* | 61 |
| *Pergamasus* sp 4 | 1 |
| *Pergamasus* sp. 3 | 11 |
| Polyaspidae, *Tracyhtes* spp. | 157 |
| Unknown sp 1 | 56 |
| Unknown sp 2 | 18 |
| Uropodidae, Sp. 2 | 18 |
| Uropodidae: sp. 1 | 280 |
| Uropodidae: sp. 3 | 15 |
| *Veigaia nemorensis* | 75 |
| *Zercon* sp 2 | 14 |
| Zercondae: *Parazercon* | 120 |
| Zerconidae: *Zercon* sp. 1 | 64 |

Supplement S4: Nematode indices

Several indices exist to describe the nematode community. In this study, we used three metrics developed by Ferris et al. (2001): the enrichment index (EI), which shows the relative importance of nematode guilds that quickly respond to nutrient enrichment, the structure index (SI), which shows the relative importance of the more persistent members of the nematode community and the channel index (CI), which shows the relative roles of fungivore versus the bacterivore energy channel in decomposition.

Nematodes are assigned a c-p (coloniser-persister) score ranging from 1 to 5 based on their ecology. Quickly colonising r-strategists with high fecundity (often bacterivores) that are able to survive harsh conditions by forming dauerlarvae, get a score of 1, while higher scores represent increasingly slow colonisers, usually larger species with lower fecundity, larger eggs and a greater sensitivity to disturbance.

To calculate ecological indices, the c-p values along with the trophic ecology of the nematode community are used, in which Ba = Bacterivore, Fu = Fungivore and Pr = Predator. A bacterivore with a c-p value of 2 is noted as Ba_2_. Based on this information, the basal (b), enrichment (e) and structure (s) components of the food web can be calculated:

$$b=({Ba}_{2}+{Fu}_{2})\times0.8$$

$$e=\left( {Ba}_{1}\times3.2 \right)+({Fu}_{2}\times0.8)$$

$$s=\left( {Ba}_{3}+{FU}_{3}+{Pr}_{3}+{Om}_{3} \right)\times1.8+\left( {Ba}_{4}+{Fu}_{4}+{Om}_{4}+{Pr}_{4} \right)\times3.2+({Pr}_{5}+{Om}_{5})\times5.0$$

With each combination of trophic ecology and c-p value representing the abundance of the respective group. Based on these values, the Enrichment index, Structure index and Channel index can then be calculated:

$$EI=100\times\frac{e}{e+b}$$

$$SI=100\times\frac{s}{s+b}$$

$$CI=100\times\frac{0.8\times{Fu}_{2}}{3.2\times{Ba}_{1}+0.8\times{Fu}_{2}}$$

Supplement S5: Model comparison

Table S5.1: Comparison of the best models from each variable category and intercept-only models, based on AICc-scores. The best model is indicated underlined and in bold.

| **Model type** | **Best model** | **AICc** | **Multiple R²** | **Adj R²** |
| --- | --- | --- | --- | --- |
| **Soil properties** |  |  |  |  |
| *C:N-ratio* |  |  |  |  |
| Null model | C:N-ratio ~ 1 | 97.0 |  |  |
| **Grazing model** | **C:N-ratio ~ Grazing** | **88.4** | **0.43** | **0.40** |
| *Bulk pH* |  |  |  |  |
| Null model | Bulk pH ~ 1 | 27.0 |  |  |
| **Grazing model** | **Bulk pH ~ Grazing** | **25.5** | **0.19** | **0.15** |
| *Olsen phosphorus* |  |  |  |  |
| Null model | log(Olsen P + 1) ~ 1 | 55.8 |  |  |
| **Grazing model** | **log(Olsen P + 1) ~ Grazing** | **43.6** | **0.53** | **0.50** |
| *Carbon content* |  |  |  |  |
| **Null model** | **Carbon content ~ 1** | **119.4** |  |  |
| Grazing model | Carbon content ~ Grazing | 121.5 | 0.04 | -0.02 |
| *Nitrogen content* |  |  |  |  |
| **Null model** | **Nitrogen content ~ 1** | **-5.9** |  |  |
| Grazing model | Nitrogen content ~ Grazing | -3.1 | 0.00 | -0.05 |
| *Soil bulk density* |  |  |  |  |
| Null model | Soil bulk density ~ 1 | -18.2 |  |  |
| **Grazing model** | **Soil bulk density ~ Grazing** | **-18.8** | **0.15** | **0.11** |
|  |  |  |  |  |
| **Vegetation** |  |  |  |  |
| *Herb layer cover* |  |  |  |  |
| Null model | log(Herb layer cover+1) ~ 1 | 45.0 |  |  |
| **Grazing model** | **log(Herb layer cover+1) ~ Grazing** | **35.2** | **0.47** | **0.44** |
| Soil model | log(Herb layer cover+1) ~ Olsen P + Soil bulk density | 38.1 | 0.47 | 0.41 |
| *Plant species richness* |  |  |  |  |
| Null model | Herb species richness ~ 1 | 129.9 |  |  |
| Grazing model | Herb species richness ~ Grazing | 125.2 | 0.31 | 0.28 |
| **Soil model** | **Herb species richness ~ Soil pH** | **117.8** | **0.53** | **0.50** |
|  |  |  |  |  |
| **Microbes** |  |  |  |  |
| *Bacterial PLFA* |  |  |  |  |
| Null model | Bacterial PLFA ~ 1 | 189.6 |  |  |
| Grazing model | Bacterial PLFA ~ Grazing | 187.6 | 0.22 | 0.17 |
| **Soil model** | **Bacterial PLFA ~ C:N-ratio** | **183.7** | **0.35** | **0.32** |
| *Fungal PLFA* |  |  |  |  |
| Null model | Fungal PLFA ~ 1 | 143.0 |  |  |
| Grazing model | Fungal PLFA ~ Grazing | 136.9 | 0.36 | 0.32 |
| **Soil model** | **Fungal PLFA ~ C:N-ratio** | **130.3** | **0.54** | 0.51 |
| *Fungal:bacterial PLFA* | |  |  |  |
| Null model | F:B-PLFA ~ 1 | -61.7 |  |  |
| **Grazing model** | **F:B-PLFA ~ Grazing** | **-75.1** | **0.56** | **0.53** |
| Soil model | F:B-PLFA ~ C:N-ratio + Olsen P | -72.3 | 0.56 | 0.51 |
|  |  |  |  |  |
| **Nematodes** |  |  |  |  |
| *Plant associated nematode abundance* | |  |  |  |
| **Null model** | **Nematodes.plant ~ 1** | **176.6** |  |  |
| Grazing model | Nematodes.plant ~ Grazing | 179.4 |  |  |
| Soil model | Nematodes.plant ~ Nitrogen content | 177.1 |  |  |
| Vegetation model | Nematodes.plant ~ Herb layer cover | 177.9 |  |  |
| *Bacterivore nematodes* | |  |  |  |
| **Null model** | **Nematodes.bact ~ 1** | **183.5** |  |  |
| Grazing model | Nematodes.bact ~ Grazing | 184.5 | 0.09 | 0.04 |
| Soil model | Nematodes.bact ~ Olsen P | 183.7 | 0.12 | 0.07 |
| Microbial model | Nematodes.bact ~ Bacterial PLFA | 186.1 | 0.01 | -0.05 |
| *Fungivore nematodes* | |  |  |  |
| Null model | Nematodes.fun ~ 1 | 178.8 |  |  |
| **Grazing model** | **Nematodes.fun ~ Grazing** | **160.4** | **0.65** | **0.64** |
| Soil model | Nematodes.fun ~ Olsen P + Soil pH | 164.3 | 0.64 | 0.60 |
| Microbial model | Nematodes.fun ~ F:B-PLFA | 168.7 | 0.48 | 0.45 |
| *Omnivorous nematodes* | |  |  |  |
| **Null model** | **Nematodes.omn ~ 1** | **162.6** |  |  |
| Grazing model | Nematodes.omn ~ Grazing | 165.3 | 0.01 | -0.05 |
| **Soil model** | Nematodes.omn ~ Soil bulk density | 165.0 | 0.02 | -0.03 |
| Prey model | Nematodes.omn ~ All other nematodes | 165.4 | 0.00 | -0.05 |
| Microbial model | Nematodes.omn ~ Fungal PLFA | 164.7 | 0.03 | -0.02 |
| *Predatory nematodes* | |  |  |  |
| Null model | Nematodes.pred ~ 1 | 151.5 |  |  |
| Grazing model | Nematodes.pred ~ Grazing | 152.8 | 0.07 | 0.02 |
| Soil model | Nematodes.pred ~ C:N-ratio | 150.9 | 0.15 | 0.11 |
| Prey model | Nematodes.pred ~ All other nematodes | 154.0 | 0.02 | -0.04 |
| **Microbial model** | **Nematodes.pred ~ F:B-PLFA** | **146.5** | **0.32** | **0.28** |
| *Nematode richness* |  |  |  |  |
| Null model | Nematode.rich ~ 1 | 121.8 |  |  |
| Grazing model | Nematode.rich ~ Grazing | 120.7 | 0.18 | 0.13 |
| **Soil model** | **Nematode.rich ~ Soil pH** | **115.7** | **0.36** | **0.32** |
| Microbial model | Nematode.rich ~ F:B-PLFA | 121.4 | 0.15 | 0.10 |
| *Enrichment index* |  |  |  |  |
| Null model | EI ~ 1 | 152.8 |  |  |
| **Grazing model** | **EI ~ Grazing** | **148.8** | **0.29** | **0.25** |
| Soil model | EI ~ Olsen P | 151.5 | 0.19 | 0.14 |
| Microbial model | EI ~ F:B-PLFA | 150.7 | 0.22 | 0.17 |
| *Structure index* |  |  |  |  |
| **Null model** | **SI ~ 1** | **149.2** |  |  |
| Grazing model | SI ~ Grazing | 151.8 | 0.01 | -0.04 |
| Soil model | SI ~ Soil pH | 151.2 | 0.04 | -0.01 |
| Microbial model | SI ~ Bacterial PLFA | 151.3 | 0.03 | -0.02 |
| *Channel index* |  |  |  |  |
| Null model | CI ~ 1 | 198.3 |  |  |
| **Grazing model** | **CI ~ Grazing** | **188.0** | **0.48** | **0.45** |
| Soil model | CI ~ Olsen P + Soil pH | 192.0 | 0.46 | 0.40 |
| Microbial model | CI ~ F:B-PLFA | 194.4 | 0.29 | 0.25 |
|  |  |  |  |  |
| **Microarthropods** |  |  |  |  |
| *Collembolan species richness* | |  |  |  |
| **Null model** | **Coll sp. rich. ~ 1** | **60.2** |  |  |
| Grazing model | Coll sp. rich. ~ Grazing | 60.5 | 0.12 | 0.07 |
| Soil model | Coll sp. rich. ~ C:N-ratio + Carbon content | 60.2 | 0.26 | 0.17 |
| Microbial model | Coll sp. rich. ~ Fungal PLFA | 62.5 | 0.03 | -0.03 |
| *Collembolan abundance* | |  |  |  |
| Null model | Coll abundance ~ 1 | 190.4 |  |  |
| Grazing model | Coll abundance ~ Grazing | 187.9 | 0.23 | 0.19 |
| **Soil model** | **Coll abundance ~ Soil pH** | **172.2** | **0.65** | **0.63** |
| Microbial model | Coll abundance ~ F:B-PLFA | 181.1 | 0.45 | 0.42 |
| *Oribatid species richness* | |  |  |  |
| Null model | Oribatid sp. rich. ~ 1 | 82.4 |  |  |
| **Grazing model** | **Oribatid sp. rich. ~ Grazing** | **75.7** | **0.38** | **0.34** |
| Soil model | Oribatid sp. rich. ~ Olsen P + Soil bulk density | 81.4 | 0.30 | 0.21 |
| Microbial model | Oribatid sp. rich. ~ F:B-PLFA | 76.6 | 0.35 | 0.31 |
| *Oribatid abundance* |  |  |  |  |
| Null model | log(Oribatid ab + 1) ~ 1 | 58.6 |  |  |
| Grazing model | log(Oribatid ab + 1) ~ Grazing | 33.8 | 0.75 | 0.73 |
| **Soil model** | **log(Oribatid ab + 1) ~ Olsen P + Soil pH + Soil BD** | **33.7** | **0.82** | **0.79** |
| Microbial model | log(Oribatid ab + 1) ~ F:B-PLFA | 44.1 | 0.58 | 0.56 |
| *Mesostigmatid species richness* | |  |  |  |
| Null model | Mesostigmata sp. rich. ~ 1 | 28.2 |  |  |
| Grazing model | Mesostigmata sp. rich. ~ Grazing | 25.3 | 0.25 | 0.21 |
| Soil model | Mesostigmata sp. rich. ~ Olsen P + Soil pH | 27.8 | 0.27 | 0.19 |
| **Prey model** | **Mesostigmata sp. rich. ~ Oribatid sp. rich.** | **24.7** | **0.27** | **0.23** |
| *Mesostigmatid abundance* | |  |  |  |
| Null model | Mesostigmata ab ~ 1 | 156.1 |  |  |
| Grazing model | Mesostigmata ab ~ Grazing | 138.3 | 0.64 | 0.62 |
| Soil model | Mesostigmata ab ~ C:N-ratio | 144.1 | 0.52 | 0.49 |
| **Prey model** | **Mesostigmata ab ~ Oribatid ab** | **127.7** | **0.79** | **0.78** |
|  |  |  |  |  |
| **Earthworms** |  |  |  |  |
| *Earthworm biomass* |  |  |  |  |
| Null model | log(Earthworm biomass + 1) ~ 1 | 56.5 |  |  |
| **Grazing model** | **log(Earthworm biomass + 1) ~ Grazing** | **51.3** | **0.33** | **0.29** |
| Soil model | log(Earthworm biomass + 1) ~ Soil pH | 56.5 | 0.13 | 0.08 |
| Microbial model | log(Earthworm biomass + 1) ~ F:B-PLFA | 57.6 | 0.08 | 0.03 |
| *Epigeic biomass* |  |  |  |  |
| Null model | Epigeic biomass ~ 1 | 31.0 |  |  |
| Grazing model | Epigeic biomass ~ Grazing | 33.7 | 0.00 | -0.05 |
| Soil model | Epigeic biomass ~ C:N-ratio | 33.0 | 0.04 | -0.02 |
| **Microbial model** | **Epigeic biomass ~ Bacterial PLFA + Fungal PLFA** | **29.0** | **0.33** | **0.25** |
| *Endogeic biomass* |  |  |  |  |
| **Null model** | **Endogeic biomass ~ 1** | **101.3** |  |  |
| Grazing model | Endogeic biomass ~ Grazing | 103.8 | 0.01 | -0.04 |
| Soil model | Endogeic biomass ~ Bulk pH + C:N-ratio | 101.3 | 0.26 | 0.18 |
| Microbial model | Endogeic biomass ~ Fungal PLFA | 103.1 | 0.05 | 0.00 |
| *Anecic biomass* |  |  |  |  |
| Null model | log(Anecic biomass + 1) ~ 1 | 55.2 |  |  |
| **Grazing model** | **log(Anecic biomass + 1) ~ Grazing** | **47.6** | **0.40** | **0.37** |
| Soil model | log(Anecic biomass + 1) ~ C:N-ratio | 49.2 | 0.36 | 0.32 |
| Microbial model | log(Anecic biomass + 1) ~ F:B-PLFA | 51.9 | 0.26 | 0.22 |

Supplement S6: Additional figures


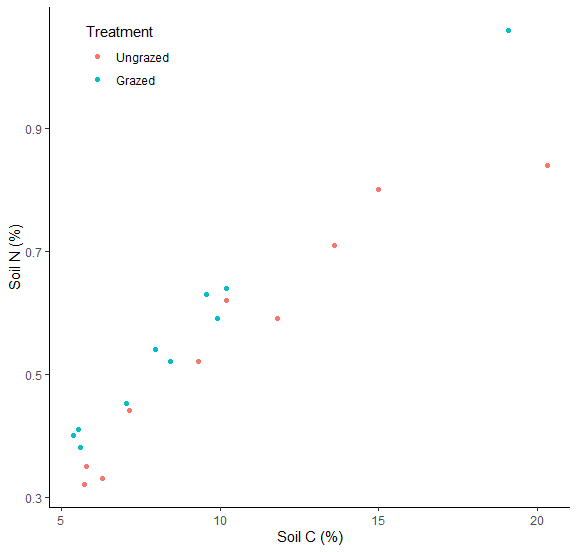


Figure S6.1: Soil carbon and nitrogen in grazed and ungrazed forest soils.


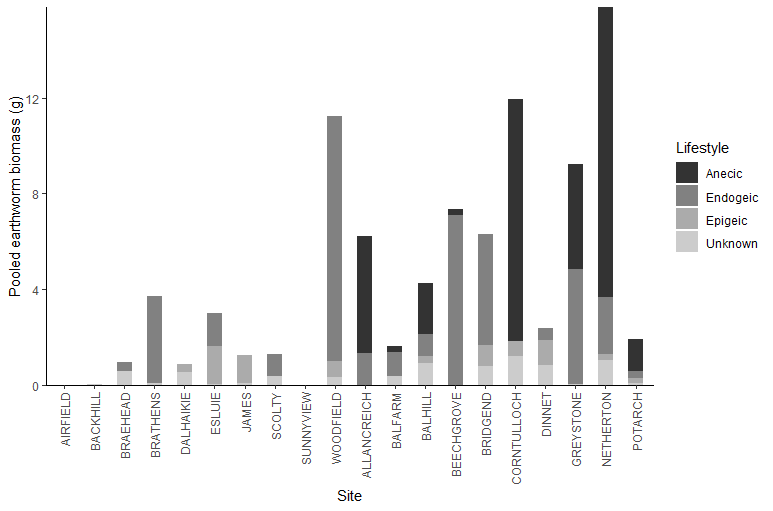


Ungrazed

Grazed

Figure S6.2: Earthworm biomass per guild, per site.
